# Towards creation of national cerebral palsy registries in Arab countries: what is missing?

**DOI:** 10.1007/s12519-021-00510-4

**Published:** 2022-02-02

**Authors:** Sahar M. A. Hassanein, Tamer A. El-Sobky

**Affiliations:** 1 Division of Pediatric Neurology, Department of Pediatrics, Faculty of Medicine, Ain Shams University, Cairo, Egypt; 2 Division of Pediatric Orthopedics, Department of Orthopedic Surgery, Faculty of Medicine, Ain Shams University, Cairo, Egypt

Cerebral palsy (CP) is a global, complex and lifelong health issue with a relatively high disease burden in low-resource countries. The benefits of a national disease registry in general and a national CP registry in particular are twofold, namely the public health and the clinical disease-related benefits. A national CP quality registry has the potential to inform health planning and spending at national, regional and continental levels. In turn, this can help allocate and manage the relevant material and human resources in a more predictable and efficient manner. Accordingly, a national CP quality registry is regarded as an important guide to strategic, health policy-making at a governmental level. In addition, national cerebral palsy registries are an invaluable source of scientific information that researchers can leverage to produce practice-changing research and high-ranking, evidence-based guidelines. These in turn can improve the quality of service delivered to patients and can upgrade treatment outcomes.

## Cerebral palsy registries in Arab countries and around the world

Australia, Scandinavia and Canada have played a leading role in the creation of national CP quality registries that direct public health planning and facilitate the establishment and growth of nationwide hip-surveillance programs. Hip dislocation in CP children is a widely prevalent and incapacitating deformity. With the benefit of such hip-surveillance programs, the number of CP children suffering from hip dislocations and requiring major bony hip salvage and reconstructive procedures has dropped dramatically [1–3]. Unfortunately, “national” cerebral palsy quality registries in the Arab world have not yet materialized. However, the first Arab initiative in this matter originated in Jordan, where a CP follow-up registry was tested in a pilot study [4]. Nevertheless, the fruits of this effort have not yet been appreciated on a national level. Several relatively small district-based CP epidemiological studies have been performed in Egypt [5–7]. Although these studies have shed some light on the prevalence and epidemiology of CP in localized geographic areas in Egypt, they are still not truly representative of the epidemiological profile of CP at a nationwide scale. Other Arab authors, especially those from Saudi Arabia, have made similar attempts to unmask the prevalence and demographics of CP [8, 9]. Encouragingly, two Egyptian registries have been established in 2014. The first is the National Registry for Egyptian Pediatric Neuromuscular Diseases (EGYPT PED-NMD) in collaboration with the international TREAT-NMD network [10]. The second registry is the National Egyptian Network Pediatric Stroke and Hemiplegia Registry (NENPSHR) in collaboration with the International Pediatric Stroke Study in Canada [11]. Developing countries, such as Bangladesh [12] and Vietnam [13], conducted pilot studies to develop a Bangladesh CP registry and a hospital-based disease surveillance program for CP children in Vietnam. Similarly, credible epidemiological research on CP children has been performed in non-Arab, African countries, such as Uganda [14].

## Non-technical barriers to creation of national CP registries in Arab countries

Contrary to common belief, the main underlying and precipitating causes of the aforementioned shortcomings are largely thought to be non-technical factors. The Arab world has a wealth of both material and human capital reserve in biomedical disciplines. However, inefficient use of the already insufficient public/institutional funds allocated to research remains an important barrier to the intensity and quality of research, including mega projects, such as establishment of national CP quality registries.

**Fig. 1:**
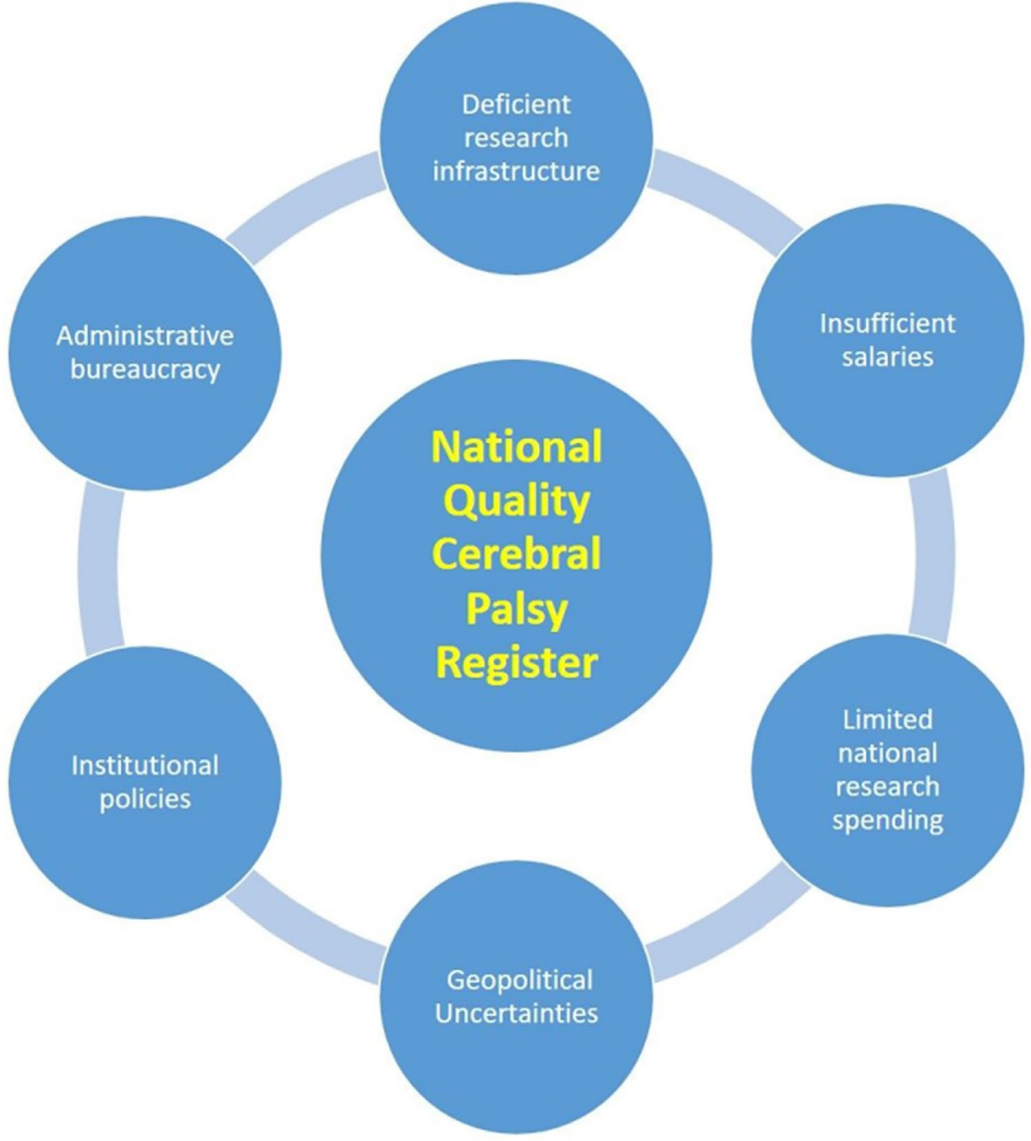
Factors impacting the establishment of national cerebral palsy registers in Arab countries

An efficient, electronic, health-information system is a fundamental pillar of a research infrastructure and is a central requirement for creating a national cerebral palsy registry. It is highly uncertain whether the current, paper-based information systems or the low-profile electronic systems present in some low-resource Arab countries are practically suited to such a mega project. This applies to both the establishment and maintenance of a national CP registry. These deficiencies of hospital information systems throw the main burden of recording, archiving, securing and retrieving research data onto the researchers. CP research is characteristically multi-disciplinary in nature, complex and data-rich with respect to both quantity and quality of the studied parameters. For example, performing research on single-event, multilevel orthopedic surgery in CP children in a developing country is challenging [15]. Such research entails an extensive amount of data recording, mining, protection and retrieval and a high standard of multi-discipline communication within the institution and occasionally at a multicenter level. The limitations of such non-technical research resources can even impact whether regular clinical trials in CP children can be executed in a time- and resource-efficient manner, let alone in a mega project like a nationwide CP quality registry. The administrative and financial support at institutional and governmental levels are equally critical to the potential success of such a project. Consequently, elimination of barriers, such as administrative bureaucracy and limited research spending, is a mandatory step for the creation of a national CP quality registry; and this is especially true in developing and low-resource Arab countries, such as Egypt (Fig. 1).

Arab researchers in general and Egyptian researchers in particular are unwillingly battling against an array of unfavorable or even deterring socioeconomic, administrative, salary-related and academic work-related environment barriers. This unfair struggle has eventually deprived all researchers from multi-disciplinary, neuromuscular teams the chance to meet their research commitments fully.

Not surprisingly, countries that lack national cerebral palsy quality registers will have a limited capacity to remain scientifically competitive and innovative, even if these countries have the most skilled researchers across the multiple disciplines of childhood disability. Therefore, the first step forward is to overcome the disabling cocktail of “para-technical” or “non-technical” barriers. Credible attempts to establish cerebral palsy registries are emerging slowly from various Arab countries. Such efforts can be regarded as a basis for constructing a nationwide quality CP registry and should be extended further through international collaboration and reliance on resource-suited and existing resources. Oil-rent/rich Arab countries should take advantage of the developed research infrastructure they possess.
